# British Institutions for the Care of the Inebriate.—II

**Published:** 1903-10-10

**Authors:** 


### HOSPITAL ADMINISTRATION.

### CONSTRUCTION AND ECONOMICS.

### BRITISH INSTITUTIONS FOR THE CARE OF THE INEBRIATE.—II.

By Our Special Medical Commissioner.

(*Continued from Vol. XXXIV., page 458.*)

THE DALRYMPLE HOME, RICKMANSWORTH.

The establishment of special institutions for the care and control of the inebriate is a movement of but recent development. The Dalrymple Home has the honour of holding the premier position as pioneer. It was originated mainly through the influence of the late Dr. Norman Kerr in 1882, assisted by the munificence of the widow of Dr. Donald Dalrymple, M.P. From 1884 to 1898 it was under the medical superintendentship of Dr. Welsh Branthwaite, now H.M. Inspector under the Inebriates Acts. Dr. Frederick S. D. Hogg, the present resident medical superintendent, was appointed in 1898. The institution is conducted by a committee elected by "The Homes for Inebriates Association," no member of the committee or of the Association deriving any pecuniary profit from the undertaking. There are two honorary consulting physicians. The home is licensed as a retreat under the Inebriates Acts, 1879–99, and is an excellent example of what may be accomplished by a philanthropically-directed enterprise.

Dalrymple Home is situate at Rickmansworth in Hertfordshire, about a mile from the station, and can be easily reached from Baker Street on the Metropolitan, or from Euston on the L. and N.W. Railway. It is a picturesque and convenient residence, delightfully placed on a finely-wooded terrace, with lawns which slope down to the river Colne, and surrounded by four-and-a-half acres of charmingly-wooded grounds. The house is commodious, and well adapted for its purpose, and the gardens, lawns, and adjacent grounds are sufficiently extensive to allow of opportunities for most forms of outdoor exercise, even a modified golf being popular. The sanitary arrangements are good, and the general conduct of the establishment excellent. We had the pleasure of dining with the inmates, and found the dietary to be well chosen, abundant, and the cooking and serving everything that could be desired. Treatment is confined to gentlemen addicted to inebriety or abuse of drugs who are themselves desirous of reformation and cure.

The home, although licensed, receives patients privately as well as "under the Act." Medical men may, we venture to think, rest satisfied that here their patients will be treated in accordance with the most rational procedures and under circumstances peculiarly fitted to re-create moral and physical vigour.

We were much struck by the evidences of efforts to secure the comfort and contentment of the patients. There are ample opportunities for amusement—a croquet lawn, an admirable asphalt tennis court, a large workshop, arrangements for net practice of cricket, an excellent billiard-room, and a small concert-room, while the reading-room is well supplied with books and newspapers, and there is also a studio and a dark room for photography. In fact in many ways Dalrymple Home may be taken as an excellent model.

The regulations and orders are reasonable and have been approved by the Secretary of State. Patients are not allowed, unless special permission is given by the Medical Superintendent to have money, blank cheque forms, or valuables in their possession; to take, or procure for themselves or others any intoxicating liquors, any drugs, or any stimulant, narcotic or sedative preparation; to leave the grounds; to enter any public house or other place where intoxicating liquors are sold; or to have meals in bedrooms. Punctual attendance at meals is strictly enjoined—breakfast at 9 A.M., luncheon at 1.30 P.M., dinner at 7 P.M., and tea at 9 P.M.

Patients retire at 10.30. A religious service is held on Sundays at 7 P.M., and unless excused by the Medical Superintendent for sickness or other sufficient reason patients must attend. Unless forbidden on medical or other grounds, smoking may be indulged in. The Home is intended only for those who are bent on restoration and infraction of discipline renders a private patient liable to expulsion or a patient under the Act may be summoned before the Justices.

The nineteenth annual report, recently issued, contains much material rich in scientific interest. Of particular value is the excellent summary of the 679 cases which have been treated since the opening of the Dalrymple Home. Of these 326 have been "under the Act," while 353 were "private" cases. Patients come from all parts of the world; but of the 679, as many as 323 have resided in the provinces; 215 came from London. As regards religion, 651 were Protestants and only 28 Roman Catholics. Most had received a good education, 165 having passed through college. The majority (345) were married, but 301 were single, and only 33 entered as widowers. Representatives from all occupations appear; we note 49 have been "medical practitioners," and 6 "students of medicine," while "gentlemen of no occupation" number 159. No history of insanity or inebriety could be obtained in 274 of the 679 cases, but a distinct history of insanity was present in 59, and inebriety in either father or mother in 121. As regards "temperament," 443 are considered to have been of "nervous" type. The returns as to associate habits are of very considerable interest: 590 were devoted to "the weed," while others indulged in chloral, morphia, cocaine, opium, chlorodyne, sulphonal, trional, bromidia, and cannabis indica. The drinking habits were designated "regular" in 479 and "periodical" in 185, but the frequency of the periodicity varied much. No fewer than 138 patients had had one attack of delirium tremens, and one patient was said to have had 13. The kind of inebriant used has varied: 189 are entered as "all spirits," 192 as whisky, 34 as brandy, 8 as gin, while in many the drinks were manifestly "mixed." The average of time addicted in all cases works out at a little over eight years. The after history is of course difficult to obtain, but of the 679, 241 are "doing well," 100 "improved," 203 still remain "not improved," 9 have become insane, 48 are dead, and of 78 it is recorded "not heard from." The Dalrymple Home is one of the very few institutions which seems imbued with the scientific spirit. It is much to be regretted that most of these retreats apparently do little towards advancing the serious study of inebriety. It would be well if those responsible for the conduct of establishments for the care of the inebriate would make themselves acquainted with the work of the Society for the Study of Inebriety, and the results of recent researches into the morbid psychology and pathological characteristics of the condition they seek to treat, in only too many cases, we fear, blindly and without guiding principles or directed merely by an unscientific empiricism born of tradition and cradled by custom.

Dr. Hogg informed us that at the Dalrymple Home the average duration of residence is about six months. Treatment is based upon sound, rational lines, free from fads and unfettered by adherence to "systems." Strict abstinence is enforced, and every means taken to increase the powers making for physical and moral resistance to the craving for drink. After three months a certain degree of liberty is allowed, but it is often abused. The ages of the patients vary from 20 to 70, but average a little above 36½ years. In about 33 per cent. of the cases favourable results are obtained. As regards ætiology, generally speaking no history is obtained of any definite "inherited craving," but frequently there is evidence of a marked family predisposition to nervous irritability. A mixed addiction, that is inebriate in alcohol and drugs, was noted in about 5 per cent. of the cases. No very definite organic disease was noted in the majority of instances, and usually after a short treatment, judged by physical signs, the greater number of the patients would pass in an examination for life assurance. It is particularly interesting to note at a time when some would contend that "alcoholic neuritis" is dependent on arsenic or toxic bodies other than alcohol that Dr. Hogg has met with most marked evidences of peripheral multiple neuritis in pure spirit drinkers. As might be expected, cases introduced "under the Act" usually do best.

The following are the stated terms: "Entrance fee £1 1s. Thirteen weeks' board and residence, in advance, from £2 2s. to £5 5s. per week, according to bedroom accommodation. Extras:—Special attendant (if required) fires in bedroom, personal laundry and medicines other than those employed for the cure of the alcohol or drug habit."

We consider the Dalrymple Home one of the best institutions at present available for the care of inebriates of moderate means and belonging to the professional and upper middle classes. We venture to think that with a little more enterprise its committee of management might do much to bring its advantages prominently before the profession, and so extend its usefulness.

(*To be continued.*)

